# The Potential Antipyretic Mechanism of Gardeniae Fructus and Its Heat-Processed Products With Plasma Metabolomics Using Rats With Yeast-Induced Fever

**DOI:** 10.3389/fphar.2019.00491

**Published:** 2019-05-09

**Authors:** Xue Zhang, Yun Wang, Shaojing Li, Yejia Dai, Xiaoqing Li, Qinghao Wang, Guoyou Wang, Yinlian Ma, Xuezhu Gu, Cun Zhang

**Affiliations:** ^1^Institute of Chinese Materia Medica, China Academy of Chinese Medical Sciences, Beijing, China; ^2^Department of Pharmacology, Anhui University of Chinese Medicine, Hefei, China; ^3^College of Pharmacy, Henan University of Chinese Medicine, Zhengzhou, China

**Keywords:** metabolomics, Gardeniae Fructus, antipyretic, processed, ultra-high-performance liquid chromatography/mass spectrometry, multivariate statistical analysis

## Abstract

Gardeniae Fructus (GF), prepared GF (GFP), and carbonized GF (GFC) are widely used in China for the treatment of fever. However, the involved antipyretic mechanism has not been fully elucidated. In this study, rectal temperature and pyrogenic cytokines were used to evaluate the antipyretic effect of raw and processed GF in rats with dry-yeast-induced pyrexia. Reverse phase and hydrophilic interaction liquid chromatography and ultra-high-performance liquid chromatography/mass spectrometry were used to acquire the metabolomics profile of GF, GFP, and GFC in rats with pyrexia. The results showed that the rectal temperature of rats treated with GF, GFP, and GFC was suppressed after 6 h (*P* < 0.05), compared with that observed in pyrexia model rats. The enzyme-linked immunosorbent assay showed that the expression of tumor necrosis factor α and interleukin 6 were suppressed by GF, GFP, and GFC. Moreover, GFC suppressed the expression of interleukin 6 significantly (*P* < 0.01). Of note, 11, 15, and 25 feature metabolites were identified in the GF, GFP, and GFC groups. Pathway analysis showed that GF mainly regulated the biosynthesis of valine, leucine, and isoleucine. Notably, GFP was involved in glycerophospholipid metabolism, while GFC was linked to glycerophospholipid and sphingolipid metabolism. These results suggested that GF, GFP, and GFC maintained their antipyretic effect despite heat processing. However, heat processing altered endogenous feature metabolites and certain pathways of GF, GFP, and GFC in rats with yeast-induced pyrexia to exert an antipyretic effect.

## Introduction

Herbal medicines are characterized by multiple components and targets in the treatment of several diseases in the clinic. Considering multiple chemical compounds involved, the pharmacological mechanism of herbs is a key factor in the development of traditional Chinese medicine (TCM). In China, GF – the dry fruit of *Gardenia jasminoides* Ellis – has been widely used in the previous two millennia for the treatment of fever, hemorrhage, and pain. GF contains various components, exerting multiple pharmacological effects (i.e., anti-inflammatory, anti-atherosclerotic, antidepressant, and neuroprotective) (Cai et al., 2015; Chen et al., 2015, 2018; Gao et al., 2015; Wang et al., 2016; Deng et al., 2018; Zang et al., 2018). Medicinal herbs are often empirically processed to improve or alter their therapeutic functions (Zhao et al., 2010). Raw and processed GF are clinically prescribed in TCM for distinct medicinal purposes (Zhao et al., 2010). GFP was produced to reduce the occurrence of side effects in the gastrointestinal tract, while the production of GFC strengthened the hemostasis effect (Zhang et al., 1994, 1995; Ling et al., 2017). All of GF, GFP and GFC could be used in the treatment of fever. However, the content of geniposide and crocin I and crocin II of GF are decreased, whereas that of tannin and crocetin of GF are increased during heat processing (Hong, 2007; Lan et al., 2014; Jian et al., 2015). Previous studies showed that heat processing changed the proportions of the components of processed GF. Whereas, the potential antipyretic effect of GF, GFP, and GFC and the mechanism involved in this process remains to be investigated.

Fever is the most commonly observed symptom caused by infection or inflammation in many diseases. Measurement of temperature permits the easy identification of a pathological condition and changes in the disease (Conti and Bartfai, 2014). Among animal models of fever, yeast-induced pyrexia in rats is the most widely used. The occurrence of pyrexia involves numerous nerval routes and factors, such as interleukin 6 (IL-6) and tumor necrosis factor alpha (TNF-α) (Luheshi, 1998). Accumulating evidences, obtained from animal model studies, suggested that the levels of TNF-α and IL-6 were increased following yeast-induced fever (Pasin et al., 2010; Dangarembizi et al., 2018). This finding implied that the levels of these factors could be used to evaluate the antipyretic effect of drugs.

Metabolomics offer the profiling of changes in the levels of multiple metabolites caused by pharmaceutical effects, by examining biofluid and tissue data and using chemometrics techniques (Nicholson et al., 2007). As we know, components of biofluids are complex and at low concentrations, which brings challenges to detection and identification for metabolomics. Ultra-high-performance liquid chromatography coupled with mass spectrometry (UHPLC/MS) is a useful method for the investigation of metabolomics due to its excellent separation and sensitivity (Chen and Singer, 2007). Furthermore, metabolomics is widely used for the “overall” chemical characterization and investigation of the mechanisms of natural herbs. Recently, this method has been applied in TCM, including formula and herb processing, etc. A previous paper used hydrophilic interaction liquid chromatography (HILIC) coupled with UHPLC/MS tandem mass spectrometry to provide a better understanding of the therapeutic effect and mechanism of Gushudan on prednisolone-induced osteoporosis in rats (Wu et al., 2018). In addition, LC/MS studies demonstrated the therapeutic effect of Danhong injection via an improvement in energy metabolism and attenuation of oxidative stress (Yi et al., 2018). Ensuring a wide metabolome coverage and enhancing the probability of biomarker detection are both essential in untargeted metabolomics studies. Moreover, the combination of HILIC and reverse phase (RP) liquid chromatography coupled with an MS detector expands the number of detected analytes and provides more comprehensive metabolite coverage (Tang et al., 2016).

**FIGURE 1:**
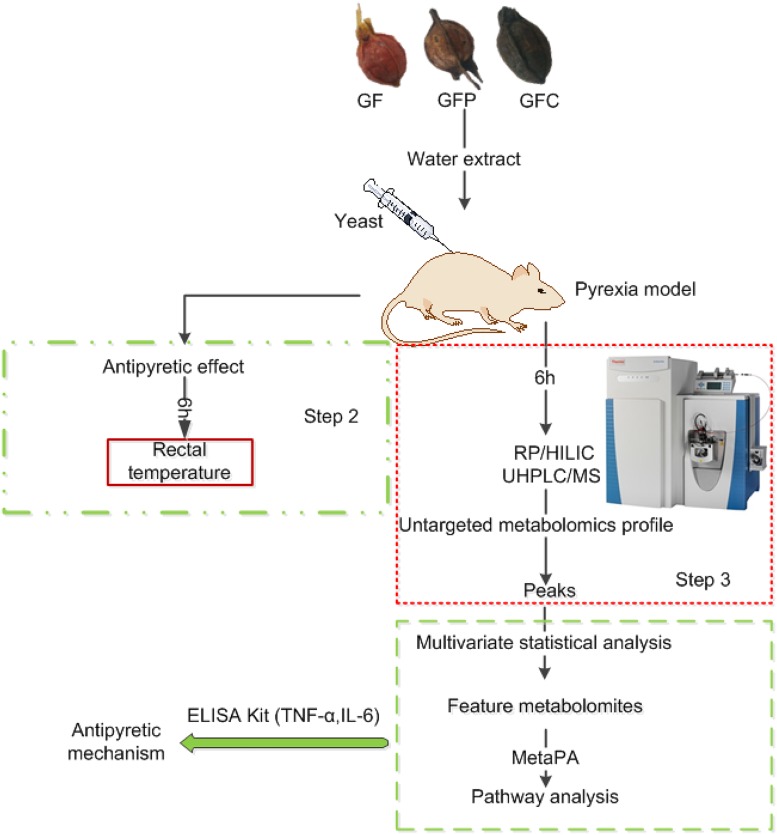
Study design for the investigation of untargeted metabolomics using hydrophilic interaction liquid chromatography (HILIC), reverse phase liquid chromatography coupled with mass spectrometry (RP/UHPLC/MS), and a yeast-induced febrile rat model to explore the antipyretic mechanism of Gardeniae Fructus (GF), prepared GF (GFP), and carbonized GF (GFC).

In the present study, we performed an untargeted metabolomics analysis using HILIC and RP UHPLC/MS to investigate the potential antipyretic effects of raw and processed GF in rats with yeast-induced fever. The design of the study is shown in Figure 1. We firstly used a rat model of yeast-induced fever and pyrogenic cytokines to assess the antipyretic effect of GF, GFP, and GFC. Secondly, following the confirmation of an antipyretic effect, we analyzed the plasma via UHPLC/MS to acquire the metabolomics profile. Thirdly, we filtered and identified feature metabolites in each group using multivariate statistics and open-access databases. Finally, we revealed the antipyretic mechanism by combining the results of the pathway analysis and enzyme-linked immunosorbent assay (ELISA).

## Materials and Methods

### Materials

#### Plant Specimens and Extract Preparation

Gardeniae Fructus, GFP, and GFC were supplied by Hebei Baicaokangshen Pharmaceutical Co., Ltd. (Hebei, China). GFP was stir-fried with the aid of a moderate fire until it became brown. GFC was stir-fried with the aid of a strong fire until it became black on the outside and brown on the inside. They were authenticated by Prof. Yongqing Xiao to be the dry fruit and processed products of *Gardenia jasminoides* Ellis, and were deposited at the Herbarium of National Resource Center for Chinese Materia Medica (CMMI), China Academy of Chinese Medical Sciences. The codes for GF, GFP and GFC specimens were CMMIYC-04639, CMMIYC-04640, and CMMIYC-04641, respectively.

GF, GFP, and GFC were extracted using boiled water (twice; 20 min per extraction). Following vacuum concentration, the extracts of GF, GFP, and GFC were converted into freeze-dried powder. The levels of gardoside, genipin 1-gentiobioside, geniposide, p-coumaroylgenipin gentiobioside, and crocins I and II in the freeze-dried powders of GF, GFP, and GFC were determined through HPLC.

#### Materials and Reagents

Gardoside, G1, G2, G3, and crocin II were separated in our laboratory, and their purities were >98%. Crocin I was supplied by Chengdu Must Bio-technology Co., Ltd. (Chengdu, China), with a purity of 99.11% (structures shown in Supplementary Figure 2). HPLC-grade acetonitrile (Thermo Fisher Scientific, Fair Lawn, NJ, United States) were used for the HPLC analysis. LC-MS-grade water, methanol, acetonitrile, and formic acid were purchased from Fisher Scientific (Loughborough, United Kingdom), while ammonium acetate purchased from Sigma-Aldrich (St. Louis, MO, United States) was used for the LC/MS analysis. Instant dry yeast was purchased from AngelYeast Co., Ltd. (Hubei, China). Chloral hydrate was purchased from Shanghai Macklin Biochemical Co., Ltd. (Shanghai, China). The ELISA kits for the rat TNF-α and IL-6 were obtained from the Nanjing Jiancheng Bioengineering Institute (Nanjing, China).

### Determination of the Levels of Iridoids and Crocins in GF and Its Heat-Processed Products

HPLC has been the most widely used method to determine the constituents of GF. GF and its heat-processed products were determined using the reported method (Li et al., 2018). A Phenomenex Luna C18 column (4.6 mm × 250 mm, 5 μm) at 35°C was used with acetonitrile (A) and 0.5% formic acid (B) at the mobile phase. The detection wavelengths were set at 254 and 440 nm. The flow rate was 1.0 ml/min. The gradient elution program proceeded as follows: 0–10 min, 6% A; 10–18 min, 6–12%A; 18–25 min, 12–17%; 25–35 min, 17–20% A; 35–45 min, 20–27% A; 45–60 min, 27–32% A; 65–70 min, 32–36% A; 70–72 min, 36–55%; 72–77 min, 55–100% A. The details are shown in Supplementary Table 1 and Supplementary Figure 1.

### Animal Model and Processing

Male Sprague-Dawley rats (170 ± 10 g) were purchased from Charles River (Beijing Vital River Laboratory Animal Technology Co., Ltd.). The animals were housed in the Good Laboratory Practice of the Chinese Academy of Chinese Medical Sciences in an isolated room maintained under the following conditions: 24 ± 1°C, 55 ± 10% relative humidity, and a 12-h light–dark cycle. The rats received water and food freely for 7 days. The rectal temperature of the rats was measured twice daily (at 9:00 a.m. and 17:00 p.m.) using a digital thermometer with a metal probe in last 3 days. The depth of rectal measurement was 3 cm and rectal temperatures with fluctuations beyond 0.5°C between the morning and evening measurements were excluded. Prior to the modeling, the rats were fasted for 12 h (free access to water). A total of 40 rats were randomly divided into five groups (*n* = 8 per group) according to weight, including one normal control (NC) group and four pyrexia model (PM) groups. The rectal temperature of all rats was measured twice prior to modeling (original temperature). Subsequently, a 15% saline suspension of yeast was injected in the back of rats (10 ml/kg) belonging to the four PM groups, whereas an equal volume of saline was injected in the animals belonging to the NC group. 5 h later, all PM groups were randomly divided into the GF group, GFP group, GFC group, and pyrexia model group (PM) according to the differences in temperature versus the original temperature (ΔT/°C). The GF, GFP, and GFC groups were administered with GF, GFP, and GFC at 4.5 g/kg (10 ml/kg) 6 h after modeling, whereas the rats in the NC and PM groups were administered with an equal volume of 0.9% saline. The rectal temperatures of rats were measured at time intervals of 1 h after administration of drugs for a total of 6 h.

This investigation was approved by the Ethics Committee on the Welfare of Laboratory Animals of the Institute of Chinese Materia Medica of the China Academy of Chinese Medical Sciences (No. 20172006). All applicable international, national, and/or institutional guidelines for the care and use of animals were followed.

### Blood Sample Collection and Preparation

Rats were anesthetized with 10% chloral hydrate (3.5 ml/kg) 6 h after treatment with GF, GFP, and GFC. Blood was collected from the rat aorta and transferred into two tubes. One of the two samples was treated with heparin sodium. Subsequently, the blood was immediately centrifuged at 3500 rpm for 15 min at 4°C and the upper plasma and serum were collected and frozen at −80°C. The plasma was used for the metabolomics analysis, while the serum was used for the cytokine assay.

Quality control samples (QCs) were prepared using a mixture of plasma samples obtained from each sample. The coefficient of variation of the ionic feature across the QCs was used to assess the stability of the analytical run. A QC filter was applied to remove any features from the analysis with a coefficient of variation >15%.

Two methods were used for HILIC and RP column separation: (1) Plasma samples and QCs were thawed on ice for 30 min. Subsequently, 300 μl menthol was added to 100 μl of plasma and vortexed for 15 s, followed by centrifugation at 14,000 × *g* for 10 min to pellet the protein. The supernatant (100 μl) was transferred for RP/UHPLC analysis; (2) The same methodology was performed, modified by the addition of acetonitrile into the plasma to pellet the protein for HILIC resolution.

### Determination of Untargeted Metabolomics Through HILIC and RP UHPLC/MS

#### Chromatography

Chromatography was performed using an UltiMate^TM^ 3000 Rapid Separation LC system (Thermo Fisher Scientific Inc., Waltham, MA, United States). The flow rate was 300 μl/min and the injection volume was 1 μl.

For RP separation, the column was an acuity UHPLC CSH C18 column (2.1 mm × 100 mm, 1.7 μm, Waters) operated at 45°C with a gradient elution program. The mobile phase A was acetonitrile/water (60/40), while the mobile phase B was isopropanol/acetonitrile (90/10); both A and B contained 0.1% formic acid and 10 mmol/l ammonium acetate. The gradient elution program was proceeded as follows: 0–1 min, 20% B; 1–11 min, 20–100% B; 11–18 min, 100% B; 1.5 min of balance back to 20% B.

For HILIC separation, a BEH Amide column (2.1 mm × 100 mm, 1.7 μm, Waters) and gradients of acetonitrile (mobile phase A) and water (mobile phase B) containing 0.1% formic acid and 10 mmol/l ammonium acetate each were used to separate the plasma samples. The column was operated at 40°C. The gradient program was set as follows: 0–1 min, 5% B; 1–7 min, 5–50% B; 7–12 min, 50% B.

#### Mass Spectrometry

The Thermo Scientific^TM^ Q Exactive^TM^ HF mass spectrometer equipped with a HESI-II probe was used for mass spectrometric detection. The positive HESI-II spray voltages were 3.7 kV, the heated capillary temperature was 320°C, the sheath gas pressure was 30 psi, the auxiliary gas setting was 10 psi, and the heated vaporizer temperature was 300°C. Both the sheath gas and the auxiliary gas were nitrogen. Data were collected in auto gain control under 1 × 10^6^ with a scan range of 150–1500 mass-to-charge (*m/z*). A maximum isolation time of 50 ms and the calibration were customized for the analysis of Q Exactive^TM^ to maintain a mass tolerance of 5 ppm. The calibrated *m/z* were 74.09643, 83.06037, 195.08465, 262.63612, 524.26496, and 1022.00341 for the positive model. Samples were analyzed in a single batch in random order with QC spectral acquisition after every six samples. The LC-MS system was controlled using the Xcalibur 2.2 SP1.48 software (Thermo Fisher Scientific), and the data were collected and processed using the same software.

### Metabolomics Statistical Analyses

The raw data were analyzed using the progenesis QI data analysis software (Non-linear Dynamics, Newcastle, United Kingdom) for peak alignment, picking, and normalization to obtain three-dimensional data containing the retention time (t_R_), *m/z*, and peak intensity for each sample. The LC/MS data were analyzed through principal components analysis (PCA) using the SIMCA-P software (version 13.0, Umetrics AB, Umeå, Sweden) to assess the main sources of variation and exclude samples beyond the confidence interval (95%). Discriminant analysis based on orthogonal partial least squares discriminant analysis (OPLS-DA) were used to filter feature metabolites with the SIMCA-P software. All data were normalized (UV-scaling) prior to constructing the OPLS-DA models. From the OPLS-DA models, values of variable importance for the projection (VIP) >1.5 indicated an important variable. Subsequently, the independent sample *t*-test was used to filter the importance variables for the NC, PM, and GF/GFP/GFC groups. Finally, the variable whose false discovery rate (FDR) was under 0.05 was chosen to be the potential biomarker.

For the identification of potential biomarkers, molecular formulae were assessed by matching accurate *m/z* measurements with metabolites via an available online database^1^^,^^2^^,^^3^. Adduct, parent, and neural ions were performed with the tolerance of the detected mass accuracy set at ±5 ppm for the identification of the candidate compounds. The adduct ions included M+H^+^, M+Na^+^, M+NH_4_^+^, 2M+H^+^, 2M+Na^+^, and 2M+NH_4_^+^. The available online databases KEGG^4^, IMPaLA version 11, and Reactome^5^ were used for the analysis of the pathway of candidate compounds.

### Serum Cytokine Assay

The content of TNF-α and IL-6 in the serum samples was quantified using ELISA kits under 450 nm wavelength according to the instructions provided by the manufacturer.

### Other Statistical Analyses

The statistical analysis of the rectal temperatures and ELISA results were performed using SPSS (version 22.0, SPSS Inc., Chicago, IL, United States). All data are presented as mean ± standard deviation except the antipyretic effect graph showing the mean ± standard error of mean. The statistical significance (*P* < 0.05 or *P* < 0.01) of differences between mean values was tested using the Student’s *t*-test.

## Results

### Antipyretic Effects

**FIGURE 2:**
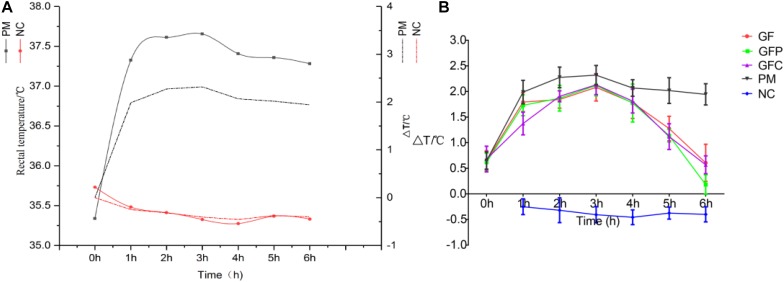
The rectal temperature of pyrexia rats and rats treated with Gardeniae Fructus (GF), prepared GF (GFP), and carbonated GF (GFC). **(A)** Rectal temperature of PM and NC. **(B)** Antipyretic effect of GF/GFP/GFC after administration (*n* = 8). Data are mean ± SEM.

The rectal temperatures of the rats at different time points were recorded to assess the pyrexia rat model versus NC rats. The rectal temperatures of PM rats were significantly increased 5 h after injection of dry yeast (Figure 2A). However, the rectal temperatures of the GF, GFP, and GFC groups were significantly suppressed on the 6 h after treatment with a dose of 4.5 g/kg (Figure 2B). An antipyretic effect was observed in the GF, GFP, and GFC groups between 4 and 6 h after treatment. The rectal temperature of rats was significantly suppressed at 5 h (*P* < 0.05) and 6 h (*P* < 0.01) (Supplementary Table 2). To explore the potential antipyretic mechanism of GF, GFP, and GFC, the plasma of rats on the 6 h after treatment was selected for subsequent metabolomics analysis.

### ELISA Results

The ELISA results (Table 1) showed that the levels of TNF-α and IL-6 in the PM group were increased compared with those observed in the NC group. The pyrexia rats treated with GF, GFP, and GFC presented a downstream effect. In the GFC group, the level of TNF-α was 32.19 ng/l with a significant difference observed compared with the PM group (^∗∗^*P* < 0.01). The levels of IL-6 in pyrexia rats treated with GF (47.51 ng/l), GFP (61.7 ng/l), and GFC (47.5 ng/l) were decreased versus those observed in the PM group (67.62 ng/l), while those of IL-6 in the GF and GFC groups were markedly decreased (^∗∗^*P* < 0.01).

**Table 1:** ELISA assay to determine the levels of TNF-α and IL-6 in the serum of rats (ng/l, *n* = 8).

	NC	PM	GF	GFP	GFC
TNF-α	48.38 ± 12.48	53.71 ± 9.93	47.45 ± 13.25	45.27 ± 6.53	32.19 ± 4.36^∗∗^
IL-6	43.37 ± 13.05	67.62 ± 10.6^Δ^	47.51 ± 6.8^∗∗^	61.7 ± 6.98	47.5 ± 6.84^∗∗^

*Data are mean ± standard deviation. ^Δ^ P < 0.01 versus normal control (NC) group; ^∗∗^P < 0.01 versus pyrexia model (PM) group.*

### Quality Control Assessment of Metabolomics Samples

**FIGURE 3:**
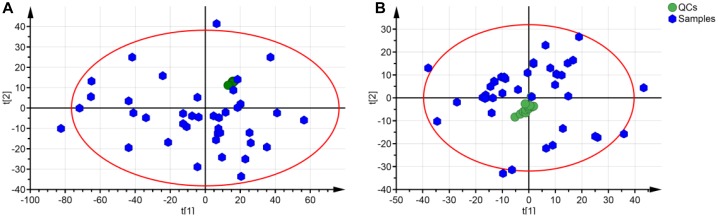
PCA plot of QCs and samples in separation mode of C18 **(A)** and HILIC **(B)**. QCs in these two separation modes were cluster together.

In the ESI positive ion scan mode, 12 QCs separated by C18 and HILIC columns were clustered together in the PCA score plots (Figure 3). Clustered QCs showed good repeatability of the separation system, and the data collected were reliable and could be further analyzed.

### Multivariate Statistical Analysis of HILIC and RP UHPLC/MS Data

**FIGURE 4:**
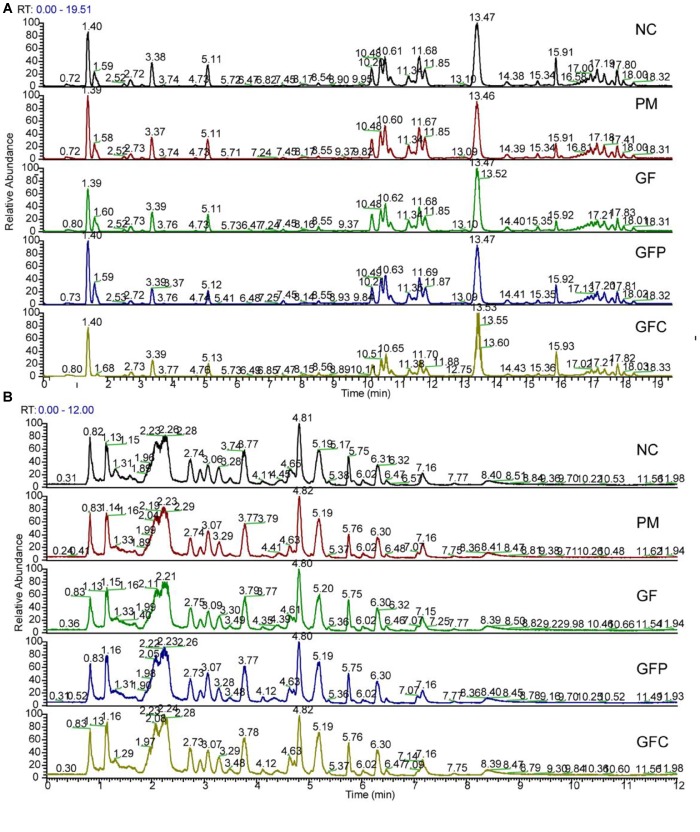
Total ion chromatograms of the normal control (NC), pyrexia model (PM), Gardeniae Fructus (GF), prepared GF (GFP), and carbonized GF (GFC) groups separated by RP UHPLC/MS **(A)** and HILIC UHPLC/MS **(B)** in the positive mode.

**FIGURE 5:**
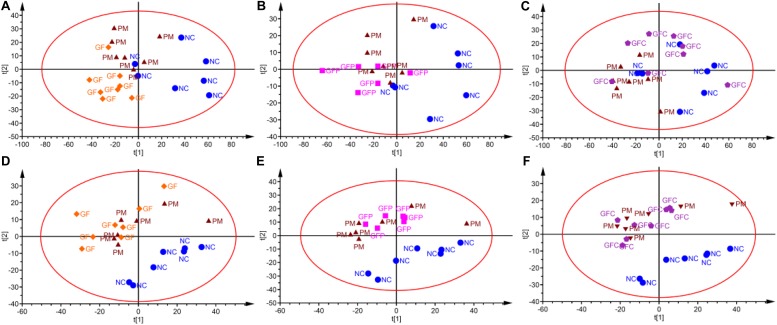
PCA scores plot of the HILIC and RP UHPLC/MS (ESI+) data evaluating variation among the NC, PM, and GF/GFP/GFC groups. PCA scores plots **(A–C)** from (NC, PM, and GF), (NC, PM, and GFP), and (NC, PM, and GFC) in RP UHPLC/MS. PCA scores plots **(D–F)** from (NC, PM, and GF), (NC, PM, and GFP), (NC, PM, and GFC) in HILIC UHPLC/MS.

Data for total ion chromatography was acquired from UHPLC/MS in the separation mode of C18 (Figure 4A) and HILIC (Figure 4B). The PCA model was used to determine the difference between the metabolite profiles among the NC, PM, and GF/GFP/GFC groups. In C18 separation, the R2 was 0.639 and Q2 was 0.415 in the NC, PM and GF groups; the R2 was 0.532 and Q2 was 0.388 in the NC, PM, and GFP groups; and the R2 was 0.64 and Q2 was 0.364 in the NC, PM, and GFC groups. The results of the PCA plot showed that the metabolite profiling of the NC, PM, and GF/GFP/GFC groups could be disguised in RP UHPLC/MS system. However, the PM and GF/GFP/GFC groups were not separated with each other through the HILIC UHPLC/MS system (Figure 5).

The OPLS-DA model and independent sample *t*-test were used to identify the ions with VIP >1.5 and FDR <0.05 as GF, GFP, and GFC groups feature metabolites, by comparing the GF, GFP, GFC groups with the PM group. Finally, 65 feature ions – the RP system contributed 22 feature ions, while the HILIC system contributed 43 marked ions – have been recalled significantly in the GF group. In addition, there were 75 feature components – the RP system contributed 11 feature ions, while the HILIC system contributed 64 marked ions – that have been called back in the GFP group. A total of 157 feature ions – the RP system contributed 140 ions, while the HILIC system contributed 17 ions – were recalled significantly in the GFC group (*P* < 0.05).

### Identification and Evaluation of Candidate Biomarkers

**FIGURE 6:**
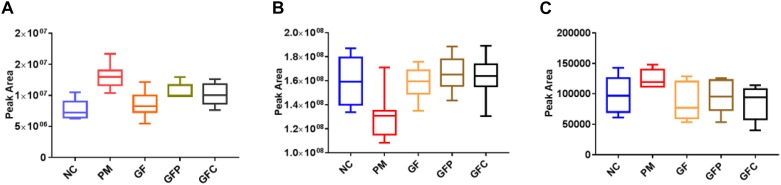
The relative content of common metabolites in raw and processed GF. **(A)** bilirubin, **(B)** LysoPC[20:4(8Z,11Z,14Z,17Z)], and **(C)** decanoylcarnitine.

According to the accurate *m/z* of ion feature metabolites, 11, 15, and 25 feature metabolites were identified in the GF, GFP, and GFC groups (Tables 2–4). The results also showed that the feature metabolites of the raw and processed GF mostly consist of phospholipids and their metabolites. Following treatment with GF, six feature metabolites were found to be phospholipids and their metabolites: LysoPC(20:5(5Z,8Z,11Z,14Z,17Z)), TG(22:2(13Z,16Z)/16:0/22:2(13Z,16Z)), glucosylceramide (d18:1/18:0), LysoPC(20:4(8Z,11Z,14Z,17Z)), LysoPC(22:2(13Z,16Z)), and LysoPC(20:4(5Z,8Z,11Z,14Z)). In the GFP group, 10 of 16 feature metabolites consisted of six lysophospholipids (LysoPCs), two phosphatidylcholines (PCs), SM(d18:0/18:1(11Z)), and PE (16:0/P-18:1(11Z)). In pyrexia rats treated with GFC, 18 of 24 metabolites consisted of five LysoPCs, seven PCs, two triglycerides (TGs), Cer(d18:1/16:0), Cer(d18:1/24:1(15Z)), Cer(d18:1/24:0), and PE (22:4(7Z,10Z,13Z,16Z)/P-18:0). The raw and processed GF has three featured metabolites, namely bilirubin, decanoylcarnitine, and LysoPC(20:4(8Z,11Z,14Z,17Z)) (Figure 6).

**Table 2:** The potential metabolites of the Gardeniae Fructus (GF) group exert a recall effect compared with the PM group: OPLS-DA model (VIP >1.5) and independent *t*-test (*P* < 0.05).

No.	t_R__*m/z*	VIP	Adduct	Formula	ppm	Metabolites	Regulation	Separated	FDR
1	1.91_542.3244	1.62	M+H^+^	C_28_H_48_NO_7_P	1	LysoPC(20:5(5Z,8Z,11Z,14Z,17Z))	↓	C18	0.0266
2	0.83_585.2698	2.05	M+H^+^	C_33_H_36_N_4_O_6_	2	Bilirubin	↓	HILIC	0.0075
3	1.06_728.6024	1.73	M+H^+^	C_42_H_81_NO_8_	2	Glucosylceramide (d18:1/18:0)	↓	HILIC	0.0286
4	1.40_316.2477	1.73	M+H^+^	C_17_H_33_NO_4_	2	Decanoylcarnitine	↓	HILIC	0.0286
5	1.95_576.4011	1.71	M+H^+^	C_30_H_58_NO_7_P	2	LysoPC(22:2(13Z,16Z))	↑	HILIC	0.0291
6	2.04_566.3203	1.90	M+Na^+^	C_28_H_50_NO_7_P	2	LysoPC(20:4(5Z,8Z,11Z,14Z))	↑	HILIC	0.0185
7	2.08_544.3386	1.73	M+H^+^	C_28_H_50_NO_7_P	2	LysoPC(20:4(8Z,11Z,14Z,17Z))	↑	HILIC	0.0286
8	3.82_248.1488	1.74	M+H^+^	C_11_H_21_NO_5_	2	Hydroxybutyrylcarnitine	↓	HILIC	0.0286
9	4.82_118.0863	1.60	M+H^+^	C_5_H_11_NO_2_	0	L-Valine	↓	HILIC	0.0430
10	5.18_229.1542	1.68	M+H^+^	C_11_H_20_N_2_O_3_	2	L-isoleucyl-L-proline	↓	HILIC	0.0313
11	11.97_136.0617	1.76	M+H^+^	C_5_H_5_N_5_	1	Adenine	↑	HILIC	0.0278

**Table 3:** The potential metabolites of the prepared GF (GFP) group exert a recall effect compared with the PM group: OPLS-DA model (VIP >1.5) and independent *t*-test (*P* < 0.05).

No.	t_R__*m/z*	VIP	Adduct	Formula	ppm	Metabolites	Regulation	Separated	FDR
1	1.91_542.3244	2.19	M+H^+^	C_28_H_48_NO_7_P	1	LysoPC(20:5(5Z,8Z,11Z,14Z,17Z))	↓	C18	0.0147
2	2.38_567.3325	2.02	M	C_30_H_50_NO_7_P	0	LysoPC(22:6(4Z,7Z,10Z,13Z,16Z,19Z))	↓	C18	0.0181
3	2.53_519.3324	2.12	M	C_26_H_50_NO_7_P	0	LysoPC(18:2(9Z,12Z))	↓	C18	0.0165
4	3.46_544.3373	1.85	M+Na^+^	C_26_H_52_NO_7_P	0	LysoPC(18:1(9Z))	↓	C18	0.0360
5	9.02_797.5792	1.86	M+NH_4_^+^	C_44_H_78_NO_8_P	1	PC (14:0/22:5(4Z,7Z,10Z,13Z,16Z))	↑	C18	0.0350
6	0.83_585.2698	1.73	M+H^+^	C_33_H_36_N_4_O_6_	2	Bilirubin	↓	HILIC	0.0489
7	1.11_830.5673	1.95	M+Na^+^	C_46_H_82_NO_8_P	0	PC (22:5(7Z,10Z,13Z,16Z,19Z)/16:0)	↑	HILIC	0.0285
8	1.37_731.6047	1.73	M+H^+^	C_41_H_83_N_2_O_6_P	2	SM(d18:0/18:1(11Z))	↓	HILIC	0.0489
9	1.40_316.2477	1.71	M+H^+^	C_17_H_33_NO_4_	2	Decanoylcarnitine	↓	HILIC	0.0494
10	1.79_719.5685	2.03	M+NH_4_^+^	C_39_H_76_NO_7_P	2	PE (16:0/P-18:1(11Z))	↓	HILIC	0.0229
11	1.95_578.4172	1.88	M+H^+^	C_30_H_60_NO_7_P	1	LysoPC(22:1(13Z))	↑	HILIC	0.0356
12	2.08_544.3386	2.00	M+H^+^	C_28_H_50_NO_7_P	2	LysoPC(20:4(8Z,11Z,14Z,17Z))	↑	HILIC	0.0241
13	3.76_227.0901	1.79	M	C_9_H_13_N_3_O_4_	2	Deoxycytidine	↓	HILIC	0.0427
14	6.89_161.1283	1.90	M+H^+^	C_7_H_16_N_2_O_2_	1	N(6)-Methyllysine	↓	HILIC	0.0339
15	7.16_129.0789	1.80	M	C_6_H_11_NO_2_	0	N-Methyl proline	↓	HILIC	0.0421

**Table 4:** The potential metabolites of the carbonized GF (GFC) group exert a recall effect compared with the PM group: OPLS-DA model (VIP >1.5) and independent *t*-test (*P* < 0.05).

No.	t_R__*m/z*	VIP	Adduct	Formula	ppm	Metabolites	Regulation	Separated	FDR
1	2.38_567.3325	1.84	M	C_30_H_50_NO_7_P	0	LysoPC(22:6(4Z,7Z,10Z,13Z,16Z,19Z))	↓	C18	0.0327
2	2.53_519.3324	1.62	M	C_26_H_50_NO_7_P	0	LysoPC(18:2(9Z,12Z))	↓	C18	0.0268
3	2.86_278.1518n	2.11	M	C_16_H_22_O_4_	0	Monoethylhexyl phthalic acid	↑	C18	0.0234
4	3.24_546.3555	2.34	M+H^+^	C_28_H_52_NO_7_P	0	LysoPC(20:3(8Z,11Z,14Z))	↑	C18	0.0234
5	7.24_284.2948	1.80	M+H^+^	C_18_H_37_NO	0	Octadecanamide	↑	C18	0.0247
6	9.30_777.5299	1.85	M	C_44_H_76_NO_8_P	1	PC (14:0/22:6(4Z,7Z,10Z,13Z,16Z,19Z))	↓	C18	0.0411
7	9.83_779.5458	1.72	M	C_44_H_78_NO_8_P	1	PC (20:4(8Z,11Z,14Z,17Z)/16:1(9Z))	↓	C18	0.0264
8	9.96_538.5195	1.68	M+H^+^	C_34_H_67_NO_3_	0	Ceramide (d18:1/16:0)	↓	C18	0.0475
9	10.87_808.5835	1.86	M+H^+^	C_46_H_82_NO_8_P	2	PC (20:4(8Z,11Z,14Z,17Z)/18:1(9Z))	↓	C18	0.0265
10	10.87_830.5632	1.90	M+Na^+^	C_46_H_82_NO_8_P	5	PC (22:5(7Z,10Z,13Z,16Z,19Z)/16:0)	↓	C18	0.0246
11	11.35_833.5917	1.70	M	C_48_H_84_NO_8_P	2	PC (22:6(4Z,7Z,10Z,13Z,16Z,19Z)/18:0)	↓	C18	0.0411
12	11.64_809.5916	1.83	M	C_46_H_84_NO_8_P	2	PC (20:1(11Z)/18:3(6Z,9Z,12Z))	↓	C18	0.0260
13	11.86_785.5925	1.68	M	C_44_H_84_NO_8_P	1	PC (18:0/18:2(9Z,12Z))	↓	C18	0.0264
14	13.91_647.6219	1.66	M	C_42_H_81_NO_3_	0	Cer(d18:1/24:1(15Z))	↓	C18	0.0327
15	14.51_780.5928	2.50	M+H^+^	C_45_H_82_NO_7_P	3	PE (22:4(7Z,10Z,13Z,16Z)/P-18:0)	↑	C18	0.0234
16	15.13_649.6374	1.52	M	C_42_H_83_NO_3_	0	Ceramide (d18:1/24:0)	↓	C18	0.0496
17	17.36_778.7043	1.55	M	C_49_H_94_O	1	TG (16:0/14:0/16:0)	↑	C18	0.0440
18	17.84_391.2839	1.74	M+H^+^	C_24_H_38_O_4_	1	Nutriacholic acid	↑	C18	0.0281
19	17.59_984.8946	1.62	M+NH_4_^+^	C_63_H_114_O_6_	1	TG(22:2(13Z,16Z)/16:0/22:2(13Z,16Z))	↓	C18	0.0281
20	0.83_585.2698	2.01	M+H^+^	C_33_H_36_N_4_O_6_	2	Bilirubin	↓	HILIC	0.0459
21	1.40_316.2477	2.17	M+H^+^	C_17_H_33_NO_4_	2	Decanoylcarnitine	↓	HILIC	0.0277
22	1.54_288.2165	2.42	M+H^+^	C_15_H_29_NO_4_	2	L-Octanoylcarnitine	↓	HILIC	0.0059
23	2.04_566.3203	1.96	M+Na^+^	C_28_H_50_NO_7_P	2	LysoPC(20:4(5Z,8Z,11Z,14Z))	↑	HILIC	0.0473
24	2.08_544.3386	2.09	M+H^+^	C_28_H_50_NO_7_P	2	LysoPC(20:4(8Z,11Z,14Z,17Z))	↑	HILIC	0.0359
25	6.89_161.1283	1.98	M+H^+^	C_7_H_16_N_2_O_2_	1	N(6)-Methyllysine	↓	HILIC	0.0468

### Pathway Analysis

**FIGURE 7:**
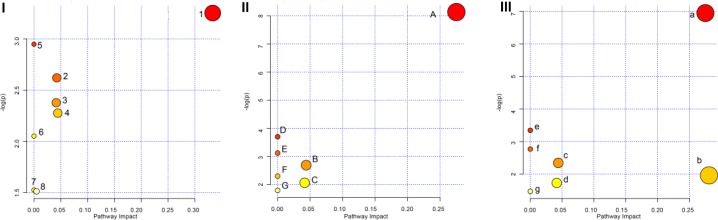
Overview of the pathway analysis for raw and processed GF. Panel **(I)** shows the pathways of GF, panel **(II)** shows the pathways of GFP, and panel **(III)** shows the pathways of GFC. (1) Valine, leucine, and isoleucine biosynthesis; (2) Porphyrin and chlorophyll metabolism; (3) Sphingolipid metabolism; (4) Glycerophospholipid metabolism; (5) Pantothenate and CoA biosynthesis; (6) Valine, leucine, and isoleucine degradation; (7) Aminoacyl-tRNA biosynthesis; (8) Purine metabolism. (A) Glycerophospholipid metabolism; (B) GPI-anchor biosynthesis; (C) Porphyrin and chlorophyll metabolism; (D) Linoleic acid metabolism; (E) alpha-Linolenic acid metabolism; (F) Sphingolipid metabolism; (G) Arachidonic acid metabolism. (a) Glycerophospholipid metabolism; (b) Sphingolipid metabolism; (c) GPI-anchor biosynthesis; (d) Porphyrin and chlorophyll metabolism; (e) Linoleic acid metabolism; (f) alpha-Linolenic acid metabolism; (g) Arachidonic acid metabolism.

The pathway analysis of GF, GFP, and GFC was performed using the KEGG IDs for each feature metabolite in their respective groups (Figure 7). The pathways regulated by all three were the glycerophospholipid metabolism, sphingolipid metabolism, and porphyrin and chlorophyll metabolism. The pathway analysis showed that GF mainly regulated the biosynthesis of valine, leucine, isoleucine, while GFP regulated the metabolism of glycerophospholipids, and GFC was involved in the metabolism of glycerophospholipids and sphingolipids (Supplementary Tables 3–5).

## Discussion

In this study, the antipyretic effect of raw and processed GF, along with untargeted metabolomics profiling based on RP and HILIC UHPLC/MS, were evaluated using a rat model of yeast-induced pyrexia. The results showed that treatment with GF, GFP, and GFC could significantly relieve fever in pyrexia rats 6 h after treatment. Subsequently, we used metabolomics to investigate the potential antipyretic mechanism of GF, GFP, and GFC through HILIC and the C18 separation mode. The PCA score plot showed that GF, GFP, and GFC failed to separate with the PM group in the HILIC UHPLC/MS system. However, they exhibited a good recall trend in the C18 UHPLC/MS system. These findings indicated that GF, GFP, GFC present an antipyretic effect focus on correcting the disturbed lipids not amide acids.

Treatment with GF resulted in reducing the level of L-valine, which was increased after the injection of yeast in rats. L-valine belongs to the branched-chain amino acid (BCAA) family which plays an important role in brain function. Previous studies showed that the levels of BCAA metabolites were enhanced as inflammatory conditions persisted (Kleber et al., 2016). Moreover, the expression of IL-10 was increased in microglial cells in response to a high concentration of BCAA (De Simone et al., 2013). Another study showed that an elevated level of BCAA in the blood could activate the NF-κB pathway and contribute to the pro-inflammatory and oxidative stress status (Zhenyukh et al., 2017). Of note, high levels of L-valine were observed in pyrexia rats with accumulated expression of IL-6 and TNF-α. Unlike GFP and GFC, GF mainly regulated the biosynthetic pathway of valine, leucine, and isoleucine. Correction of the level of valine may prevent the expression of inflammatory cytokines, leading to an antipyretic effect. The amount and types of feature metabolites belonging to lipids increased in heat-processed GF products, especially for GFC. After processing, the main pathways of GFP and GFC were altered due to the regulation of different lipids. GFP mainly regulated the metabolism of glycerophospholipids, while GFC was involved in the metabolism of glycerophospholipids and sphingolipids.

Moreover, the feature metabolites of LysoPCs and PCs in GF, GFP, and GFC closely related to prostaglandin E2 (PGE_2_). PGE_2_ is the vital substance that induces pyrexia (Wang and Qian, 2015). In the process of PGE_2_ synthesis, the phospholipids of the cell membrane generate arachidonic acid with phosphatidase 2, further generating PGE_2_ under the action of cyclooxygenase 2 (COX_2_). LysoPCs could also generate PCs with lysophosphatidylcholine acyltransferase (LPCAT). In the present study, we found that the level of PCs and LysoPC(20:5(5Z,8Z,11Z,14Z,17Z)) were significantly down-regulated in pyrexia rats treated with GF, GFP, and GFC. This effect may suppress the expression of PGE_2_, consequently relieving fever.

**FIGURE 8:**
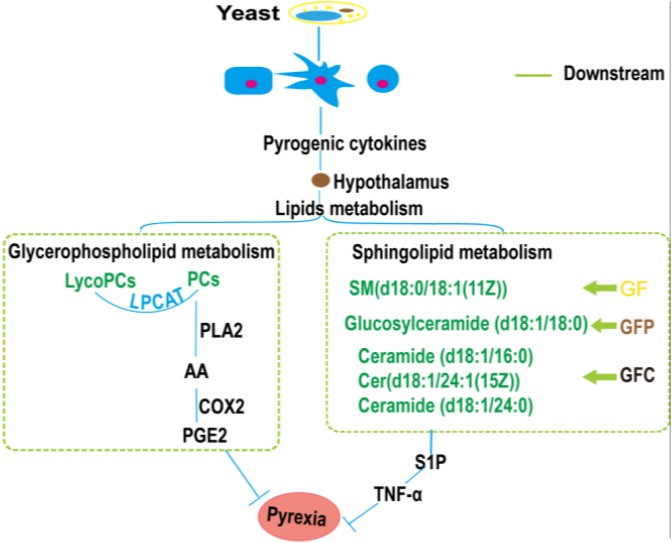
The pathways closely associated with the antipyretic effect of Gardeniae Fructus (GF), prepared GF (GFP), and carbonized GF (GFC).

Sphingolipids metabolites are an important class of lipids that play vital roles in inflammatory signaling. The main sphingolipids include SM, Cer, sphingosine (Sph), sphingosine-1-phosphate (S1P), and glucosylceramide (GluCer) (Grosch et al., 2018). Cer, C1P, and S1P are vital for the signaling of inflammatory pathways. Previous studies showed that the pathway involved in yeast-induced fever in rats mainly included the glycerophospholipid metabolism, sphingolipid metabolism, valine, leucine, and isoleucine biosynthesis and purine metabolism, etc. (Gao et al., 2014; Liu et al., 2016). Evidence has shown that TNF-α was orchestrated by sphingolipid metabolites (Kolesnick and Golde, 1994) and played an important role in apoptosis (Ohta et al., 1994). In our research, the level of glucosylceramide (d18:1/18:0) in the GF group, SM(d18:0/18:1(11Z)) in the GFP and Cer(d18:1/16:0) in the GFC group were obviously down-regulated. Consequently, the expression of S1P could be suppressed to inhibit the inflammatory pathway generating pyrogenic cytokines (Figure 8).

Furthermore, the ELISA showed that the levels of IL-6 and TNF-α in the GF, GFP, and GFC groups were decreased versus those observed in the PM group. Evidence showed that the mRNA levels of IL-6 and TNF-α increased in rats with yeast-induced fever (Dangarembizi et al., 2018). It has been shown that IL-6 can act in the brain to cause fever (Harden et al., 2008). A clinical case report found that inhibition of IL-6 expression could relieve familial Mediterranean fever (Stein et al., 2013). IL-6 operates downstream of IL-1 in the median periotic nucleus region of the hypothalamus to induce the synthesis of COX_2_. This enzyme is responsible for the production of additional PGE_2_ – the major pyrogenic mediator of fever (Evans et al., 2015). These results showed that raw and processed GF exerted an antipyretic effect, probably through the inhibition of PGE_2_ and IL-6 expression.

Consistent with the suggestions of previous studies, GF may down-regulate the level of valine to prevent the activation of the NF-κB pathway and suppress the expression of inflammatory cytokines. Moreover, GF and its heat-processed products may inhibit the expression of LycoPCs, PCs, and sphingolipids, which belong to glycerophospholipid metabolism and sphingolipids metabolism, to suppress the expression of TNF-α and IL-6. Furthermore, inhibition of IL-6 and TNF-α expression may relieve fever. The present study confirmed this hypothesis, showing that GFC effectively inhibited the expression of TNF and IL-6. Meanwhile, it regulates a larger number of lipids compared with GF and GFP.

## Conclusion

Treatment with GF, GFP, and GFC maintained their antipyretic effect after heat processing. However, heat processing altered endogenous feature metabolites and certain pathways involved in the antipyretic effect of GF, GFP, and GFC. GF mainly regulated the biosynthesis of valine, leucine, and isoleucine, while GFP regulated the metabolism of glycerophospholipids, and GFC was involved in the metabolism of glycerophospholipids and sphingolipids. Moreover, treatment with GF, GFP, and GFC suppressed the expression of TNF-α and IL-6, which are closely related to the metabolism of glycerophospholipids and sphingolipids.

## Ethics Statement

This investigation was approved by the Ethics Committee on the Welfare of Laboratory Animals of Institute of Chinese Materia Medica of China Academy of Chinese Medical Sciences (No. 20172006). All applicable international, national, and/or institutional guidelines for the care and use of animals were followed.

## Author Contributions

XZ and YW conceived and designed the experiments. XZ wrote the manuscript and analyzed the data. CZ and YW guided the experiments. SL, CZ, and YW revised the manuscript. XZ, YD, XL, QW, GW, YM, and XZ performed this experiments. CZ acquired funding for the research. All authors read and approved the final version of the manuscript.

## Conflict of Interest Statement

The authors declare that the research was conducted in the absence of any commercial or financial relationships that could be construed as a potential conflict of interest.

## Footnotes

1 http://www.hmdb.ca/

2 https://metlin.scripps.edu

3 http://www.lipidmaps.org/

4 http://www.genome.jp/kegg/

5 https://reactome.org/

## Abbreviations

GF: Gardeniae Fructus
GFC: carbonized Gardeniae Fructus
GFP: prepared Gardeniae Fructus.
