# Supplementary material for: The Potential Antipyretic Mechanism of Gardeniae Fructus and Its Heat-Processed Products With Plasma Metabolomics Using Rats With Yeast-Induced Fever
Source: Front Pharmacol. 2019 May 9;10:491. doi: 10.3389/fphar.2019.00491 (PMC6521858; doi:10.3389/fphar.2019.00491)
Supplement: Supplementary file 1 [file Table_1.DOC]

**The potential antipyretic mechanism of Gardeniae Fructus and its heat processed products with plasma metabolomics using rats with yeast-induced fever**

Zhang Xue1,[[1]](#footnote-2)#, Wang Yun^1,#^, Li Shao-jing^1^, Dai Ye-jia^1,2^, Li Xiao-qing^1,3^, Wang Qing-hao^1,3^, Wang Guo-you^1,3^, Ma Yin-lian^1^,Gu Xue-zhu^1^, Zhang Cun^1,2,3*^

^1^ Institute of Chinese Materia Medica, China Academy of Chinese Medical Sciences, Beijing, China

^2^ College of Pharmacy, Anhui University of Chinese Medicine, Hefei, China

^3^ College of Pharmacy, Henan University of Chinese Medicine, Zhengzhou, China

**Determination of iridoids and crocins of GF and its heat processed products**

To analyse the major active constituents and assess the quality of Gardeniae Fructus (GF) and its heat processed products, they were analysed by HPLC. The typical chromatograms of the GF and its heat processed products were presented in Figure 1. Six peaks of GF were identified by chemical standards which were gardoside; genipin 1- gentiobioside (G1), geniposide (G2), p-coumaroylgenipin gentiobioside (G3), crocin I and crocin II (structure shown in Figure 2). The results showed the contents of six constituents of GF, GFP and GFC were decreased in the heat process. Crocins were decreased sharply by the heat process and crocin II were failed to determination in GFC (Table 1).

Table 1 The content of six constituents among GF and its heat processed products with HPLC (mg/g).

|  | GF | GFP | GFC |
| --- | --- | --- | --- |
| Gardoside | 7.56 | 2.41 | 1.67 |
| G1 | 23.51 | 24.93 | 16.71 |
| G2 | 46.61 | 52.10 | 29.75 |
| G3 | 12.53 | 10.63 | 5.38 |
| Crocin I | 8.05 | 0.39 | 0.05 |
| Crocin II | 0.34 | 0.02 | - |

Note: ‘-’ stands for constituent failed to quantity.


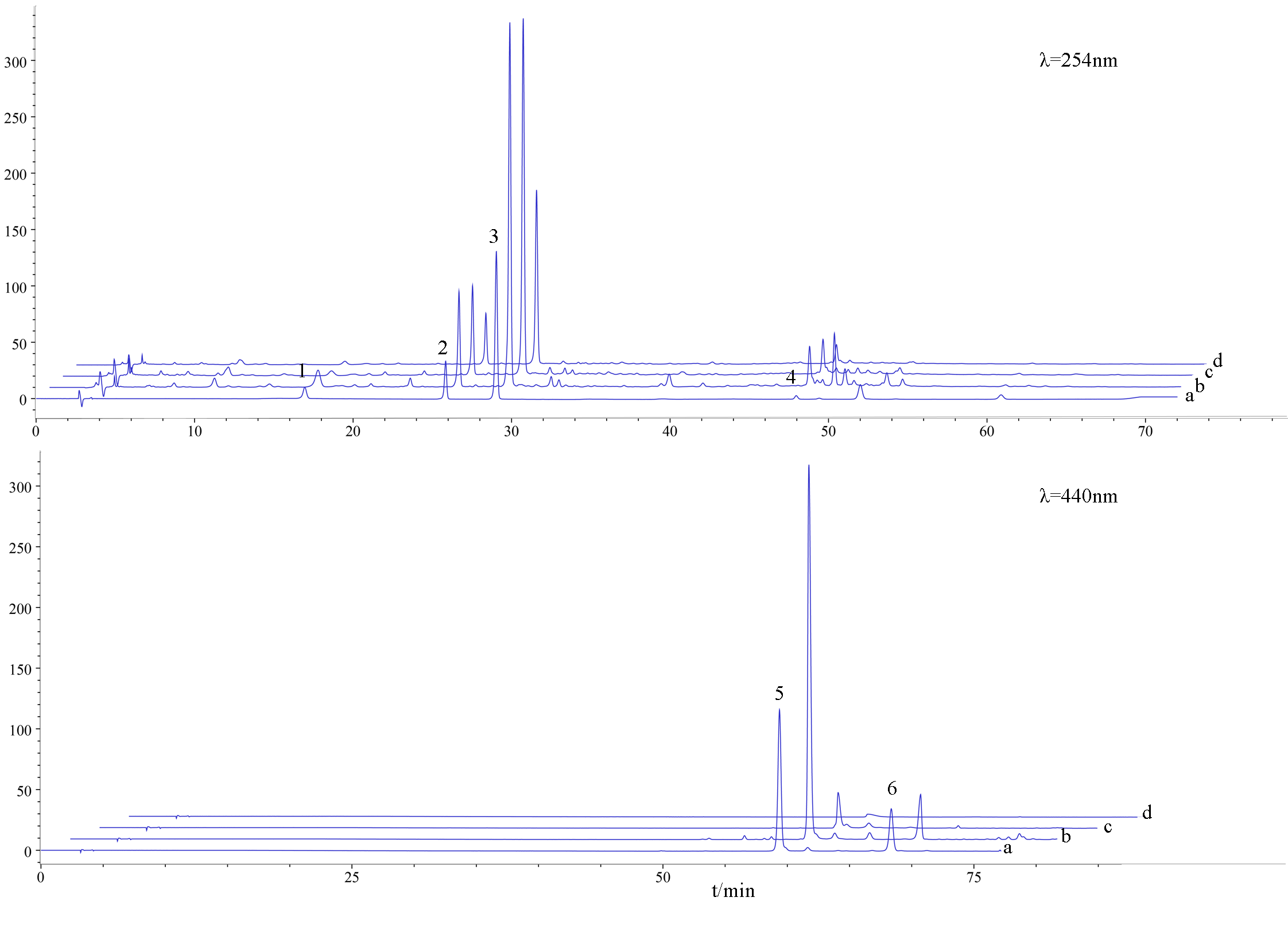


Figure 1 Typical chromatographs of GF, GFP and GFC. In figure 1 a was mixed reference standards with six determined constituents, b was Gardenia Fructus (GF), c was prepared GF, d was carbonized GF (GFC). In figure 1 the number of 1 was gardoside, 2 was genipin 1- gentiobioside, 3 was geniposide, 4 was p-coumaroylgenipin gentiobioside, 5 was concin I and 6 was concin II. Iridoids were detected under 254nm and crocins were determined under 440nm.

**gardoside**: (1S,4aS,6S,7aS)-6-hydroxy-7-methylidene-1-[(2S,3R,4S,5S,6R)-3,4,5-trihydroxy-6-(hydroxymethyl)oxan-2-yl]oxy-4a,5,6,7a-tetrahydro-1H-cyclopenta[c]pyran-4-carboxylic acid

**genipin 1- gentiobioside**: methyl (1S,4aS,7aS)-7-(hydroxymethyl)-1-[(2S,3R,4S,5S,6R)-3,4,5-trihydroxy-6-[[(2R,3R,4S,5S,6R)-3,4,5-trihydroxy-6-(hydroxymethyl)oxan-2-yl]oxymethyl]oxan-2-yl]oxy-1,4a,5,7a-tetrahydrocyclopenta[c]pyran-4-carboxylate

**geniposide**: methyl (1S,4aS,7aS)-7-(hydroxymethyl)-1-[(2S,3R,4S,5S,6R)-3,4,5-trihydroxy-6-(hydroxymethyl)oxan-2-yl]oxy-1,4a,5,7a-tetrahydrocyclopenta[c]pyran-4-carboxylate

**p-coumaroylgenipin gentiobioside**: (1S,4aS,7aS)-7-(hydroxymethyl)-1-[(2S,3R,4S,5S,6R)-3,4,5-trihydroxy-6-[[(2R,3R,4S,5S,6R)-3,4,5-trihydroxy-6-[[(E)-3-(4-hydroxyphenyl)prop-2-enoyl]oxymethyl]oxan-2-yl]oxymethyl]oxan-2-yl]oxy-1,4a,5,7a-tetrahydrocyclopenta[c]pyran-4-carboxylic acid

**crocin I**: bis[(2S,3R,4S,5S,6R)-3,4,5-trihydroxy-6-[[(2R,3R,4S,5S,6R)-3,4,5-trihydroxy-6-(hydroxymethyl)oxan-2-yl]oxymethyl]oxan-2-yl] (2E,4E,6E,8E,10E,12E,14E)-2,6,11,15-tetramethylhexadeca-2,4,6,8,10,12,14-heptaenedioate

**crocin II:** 1-O-[(2R,3R,4S,5S,6R)-3,4,5-trihydroxy-6-(hydroxymethyl)oxan-2-yl] 16-O-[(2S,3R,4S,5S,6R)-3,4,5-trihydroxy-6-[[(2R,3R,4S,5S,6R)-3,4,5-trihydroxy-6-(hydroxymethyl)oxan-2-yl]oxymethyl]oxan-2-yl] (2E,4E,6E,8E,10E,12E,14E)-2,6,11,15-tetramethylhexadeca-2,4,6,8,10,12,14-heptaenedioate


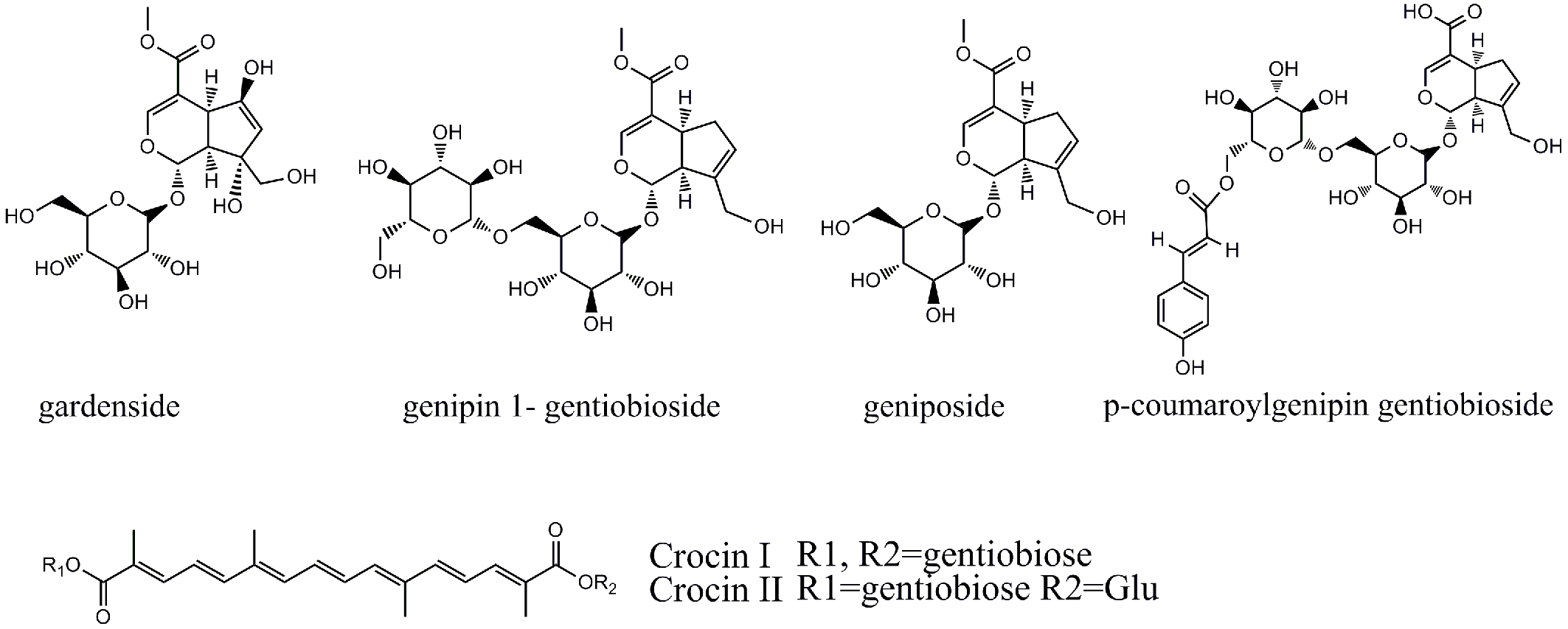


Figure 2 The structures of constituents were determined in this study.

**Antipyretic effects**

GF, GFP and GFC showed the antipyretic effect from 4 to 6 hours after they were administrated to rats, and the rectal temperature of rats were suppressed significantly on the fifth hour (*P*<0.05) and the sixth hour (*P*<0.01) after they treated with GF, GFP and GFC (Table 2).

Table 2 Antipyretic effect of GF/GFP/GFC after administrated (△T/℃, n=8）

| Group | 1h | 2h | 3h | 4h | 5h | 6h |
| --- | --- | --- | --- | --- | --- | --- |
| NC | -0.25±0.44 | -0.32±0.68 | -0.41±0.45 | -0.46±0.41 | -0.38±0.33 | -0.4±0.42 |
| PM | 1.99±0.65^△^ | 2.28±0.57^△^ | 2.32±0.53^△^ | 2.07±0.46^△^ | 2.02±0.71^△^ | 1.94±0.59^△^ |
| GF | 1.79±0.53 | 1.84±0.48 | 2.08±0.75 | 1.78±0.87 | 1.28±0.68^*^ | 0.61±1.03^**^ |
| GFP | 1.73±0.58 | 1.87±0.71 | 2.12±0.58 | 1.78±1.05 | 1.14±0.35^**^ | 0.18±0.54^**^ |
| GFC | 1.38±0.63 | 1.91±0.3 | 2.13±0.54 | 1.81±0.66 | 1.12±0.71^*^ | 0.57±0.49^**^ |

Values were presented as mean±standard deviation, ^△^*P*<0.01 compared with control group through independent sample t test and ^*^*P*<0.05 and ^**^ *P*<0.01 compared with PM group by independent sample t test.

**Pathway analysis**

Pathway analysis showed that GF mainly regulated valine, leucine and isoleucine biosynthesis, GFP centered on glycerophospholipid metabolism, and GFC focused on glycerophospholipid metabolism and sphingolipid metabolism. Meanwhile, they also regulated other pathways shown in Table 3~5.

Table 3 Summary of pathway analysis with MetaboAnalyst 4.0 of GF group.

| Pathway Name | Total | Expected | Hits | Raw p | Impact | -LOG(p) |
| --- | --- | --- | --- | --- | --- | --- |
| Valine, leucine and isoleucine biosynthesis | 11 | 0.0392 | 1 | 0.0387 | 0.3333 | 3.2526 |
| Pantothenate and CoA biosynthesis | 15 | 0.0535 | 1 | 0.0524 | 0.0000 | 2.9482 |
| Sphingolipid metabolism | 21 | 0.0749 | 1 | 0.0728 | 0.0426 | 2.6203 |
| Porphyrin and chlorophyll metabolism | 27 | 0.0963 | 1 | 0.0928 | 0.0415 | 2.3775 |
| Glycerophospholipid metabolism | 30 | 0.1070 | 1 | 0.1027 | 0.0444 | 2.2764 |
| Valine, leucine and isoleucine degradation | 38 | 0.1355 | 1 | 0.1285 | 0.0000 | 2.0515 |
| Aminoacyl-tRNA biosynthesis | 67 | 0.2389 | 1 | 0.2175 | 0.0000 | 1.5258 |
| Purine metabolism | 68 | 0.2425 | 1 | 0.2204 | 0.0047 | 1.5124 |

Table 4 Summary of pathway analysis with MetaboAnalyst 4.0 of GFP group.

| Pathway Name | Total | Expected | Hits | Raw p | Impact | -LOG(p) |
| --- | --- | --- | --- | --- | --- | --- |
| Glycerophospholipid metabolism | 30 | 0.1712 | 3 | 0.0005 | 0.2750 | 7.6816 |
| Linoleic acid metabolism | 5 | 0.0285 | 1 | 0.0282 | 0.0000 | 3.5668 |
| alpha-Linolenic acid metabolism | 9 | 0.0514 | 1 | 0.0503 | 0.0000 | 2.9890 |
| GPI-anchor biosynthesis | 14 | 0.0799 | 1 | 0.0773 | 0.0439 | 2.5596 |
| Sphingolipid metabolism | 21 | 0.1198 | 1 | 0.1140 | 0.0000 | 2.1715 |
| Porphyrin and chlorophyll metabolism | 27 | 0.1541 | 1 | 0.1444 | 0.0415 | 1.9351 |
| Arachidonic acid metabolism | 36 | 0.2054 | 1 | 0.1883 | 0.0000 | 1.6697 |

Table 5 Summary of pathway analysis with MetaboAnalyst 4.0 of GFC group.

| Pathway Name | Total | Expected | Hits | Raw p | Impact | -LOG(p) |
| --- | --- | --- | --- | --- | --- | --- |
| Glycerophospholipid metabolism | 30 | 0.2140 | 3 | 0.0001 | 0.2750 | 6.9486 |
| Linoleic acid metabolism | 5 | 0.0357 | 1 | 0.0352 | 0.0000 | 3.3465 |
| alpha-Linolenic acid metabolism | 9 | 0.0642 | 1 | 0.0626 | 0.0000 | 2.7715 |
| GPI-anchor biosynthesis | 14 | 0.0999 | 1 | 0.0958 | 0.0439 | 2.3457 |
| Sphingolipid metabolism | 21 | 0.1498 | 1 | 0.1405 | 0.2807 | 1.9625 |
| Porphyrin and chlorophyll metabolism | 27 | 0.1926 | 1 | 0.1772 | 0.0415 | 1.7302 |
| Arachidonic acid metabolism | 36 | 0.2568 | 1 | 0.2297 | 0.0000 | 1.4710 |

1. ^^ Zhang Xue and Wang Yun contributed equally.

   ^*^ To whom correspondence should be addressed.

   E-mail [zhc95@163.com](mailto:zhc95@163.com) (Zhang Cun) [↑](#footnote-ref-2)
